# Recycling of e-waste power cables using microwave-induced pyrolysis – process characteristics and facile recovery of copper metal

**DOI:** 10.1039/d4ra05602g

**Published:** 2024-09-20

**Authors:** Satoshi Horikoshi, Naoki Hachisuga, Nick Serpone

**Affiliations:** a Department of Materials and Life Sciences, Sophia University 7-1 Kioi-cho Chiyoda-ku Tokyo 102-8554 Japan horikosi@sophia.ac.jp; b PhotoGreen Laboratory, Dipartimento di Chimica, Università di Pavia Via Taramelli 12 Pavia 27100 Italy

## Abstract

This article reports on recycling e-wastes using a VVF power cable as a model through a rapid pyrolytic process following exposure to microwave radiation. This occurred *via* three possible pathways: (i) discharges at the copper wire on exposure to microwaves, with heat produced causing the thermal decomposition of the covering material – a relationship exists between the length of the copper wire and the wavelength of the microwaves; (ii) microwave heating softened the wire's covering material and ultimately led to its decomposition – in addition, the coating material carbonized by the discharge is rapidly heated by microwaves; (iii) the carbonaceous component present in the covering material absorbed the microwaves, causing the thermal decomposition. On the other hand, for VVF cables longer than 12 cm canceled the wavelength-dependent process, and the longer the VVF cable was, the more efficient was the microwave-induced pyrolysis, therefore eliminating the need to pre-cut the waste VVF cable into smaller pieces. The microwave-induced pyrolysis showed that chlorine could be recycled as HCl and the carbon and activated carbon produced could be recovered as carbon black. While conventional pyrolysis might produce tar substances and polycyclic aromatic compounds, microwave pyrolysis has been shown to enable extremely rapid resource recovery, with only C_6_ to C_12_ linear alcohols produced as intermediates; no formation of tar-like substances, polycyclic aromatic compounds, or dioxins were detected. Clearly, microwave-induced pyrolysis has proven suitable for recycling/recovery of e-waste containing metals and requires no pre-treatment to separate the plastics from the metals.

## Abstract 2

Recycling e-wastes using a VVF power cable through a rapid pyrolytic mechanism by microwave radiation is reported, and it is possible to produce copper wire and carbon resources without going through highly toxic intermediates.

## Graphical abstract

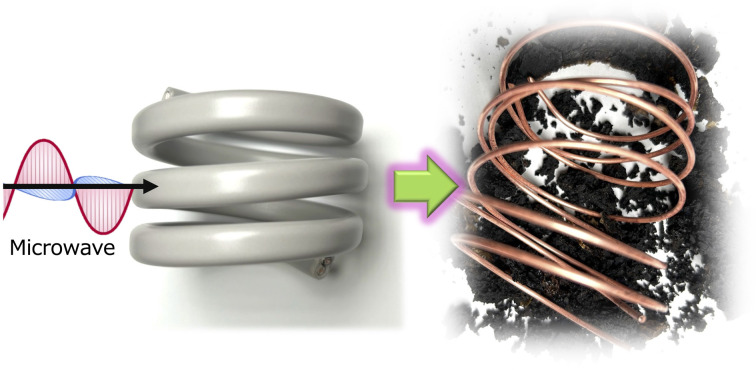

## 1 Introduction

Electronic waste, commonly referred to as e-waste, includes all discarded electrical and electronic equipment (EEE) and their components. It encompasses items that have been disposed of as waste without the intention of recycling and re-use.^1^ E-waste is also known as WEEE (Waste Electrical and Electronic Equipment), electronic waste, or e-scrap in various regions and contexts. The increasing use of electronic devices has raised concerns about the generation and management of electronic waste.^2,3^ In this regard, a staggering 62 million tons of e-waste was produced in 2022, marking an 82% increase from 2010, and projected to rise by another 32% (to approximately 82 million tons) by 2030. Valuable resources worth billions of dollars are continuously squandered and discarded. Only around 1% of the demand for rare earth elements is met through e-waste recycling.^4^ Additionally, in 2022, less than one quarter (22.3%) of the e-waste generated was properly collected and recycled, leading to approximately US $62 billion worth of recoverable natural resources going unaccounted for. This has resulted in widespread pollution and environmental concerns for communities. The practice of exporting e-waste from developed countries to developing countries has made e-waste management a critical issue that affects the latter's environment and public health.

The current value of e-waste lies in the recovery of valuable metals, semiconductors, as well as rare and precious metals. Adopting an ecosystem approach is encouraged as a sustainable and environmentally friendly way to plan for e-waste disposal and the reclamation of noble metals.^5^ However, the construction of large-scale eco-friendly processes is not fashionable in many places. Rather, concentrated acids, alkalis, and cyanide are being used during casual metal recovery, which can lead to severe human health issues as well as environmental hazards.^6^ Because of poor or disrupted engineered infrastructure and costly management processes, most countries currently manage their e-waste nonchalantly without safe metal recovery techniques.^7,8^ In a significant development in e-waste news, the European Parliament recently passed a bill (October 2022) to standardize the charging connectors of electronic devices to “USB Type-C”. This initiative will significantly contribute to reducing resources and waste. Such efforts are likely to spread globally, particularly in Europe. However, because of the high rate at which e-waste is generated, new processes for chemical resourcing of e-wastes are undeniably needed.

Electrical wiring cables, made of copper or aluminium wires, are a significant waste problem. Despite being mostly unseen by consumers, these cables are highly recyclable due to their materials. The cables' structure does not vary significantly based on their intended use. PVC is commonly used as the insulation material for electric cables due to its excellent insulating and flame-retardant properties. The process of disassembling cables involves removing the PVC insulation material using a wire stripper.^9^ While the machine's operation principle is simple, the process can be time-consuming depending on the equipment type and the amount of scrap. After removing the PVC covering, the metals are separated from the materials and recycled.

The recycling rate for PVC insulation material is currently only about 35%, with the majority ending up in landfills.^10^ Since the calorific value of PVC combustion is 4500 kcal kg^−1^, there is a hope to utilize it as an energy resource.^10^ Thermal recycling has been considered for PVC covering materials, but it generates CO_2_ and is currently not suitable for recycling PVC insulation materials. However, research is underway for the re-use of stripped PVC covering material,^11^ although this is largely limited to local processes. Past research has explored methods such as recovering fuel oil through low temperature pyrolysis and treating PVC waste in high-temperature water.^12^ Currently, treatment facilities produce significant amounts of WEEE residues, primarily consisting of mixed polymeric materials, which cannot be effectively recovered using conventional methods. Consequently, a novel pyrolysis process could potentially offer an alternative solution to eliminating these residues in landfill sites.

The microwave pyrolysis method could be a promising way to recycle the leftover materials from current recycling processes. Previous studies have looked into using microwaves to aid in the recycling of WEEEs. For example, Andersson and colleagues^13^ highlighted how different WEEEs can influence the pyrolysis process and the reduction in mass when using an experimental microwave pyrolysis reactor. Nearly a decade ago, Risco and co-workers^14^ conducted a systematic review on the chemical recovery of electrical and electronic equipment wastes through microwave-assisted pyrolysis. However, there are few research examples on this topic. One reason for this is that while microwaves do not rely on thermal conduction, many plastics have low microwave absorption, requiring high-power microwave irradiation to carry out the pyrolysis. This can be observed when heating food in a microwave oven, as the plastic container remains cool to the touch. However, e-wastes are not made up of pure plastics, as they contain mixtures that may include a plasticizer (*e.g.* dioctyl phthalate), a filler (*e.g.* calcium carbonate), and a stabilizer, in addition to metals and semiconductor components.^14^ Due to this complexity, it is important to understand the characteristics of e-waste pyrolysis through microwave radiation and to identify the strengths and weaknesses of microwave pyrolysis, while also suggesting potential optimization factors.

In this study, we used VVF cables as a model of e-waste, which are commonly used as power cables in homes and buildings. We investigated the pyrolysis behaviour of these cables using 100–300 W microwaves and sought to clarify the cause and mechanism of this pyrolysis behaviour.

## 2 Experimental section

### 2.1 E-waste model and experimental methods

**Fig. 1:**
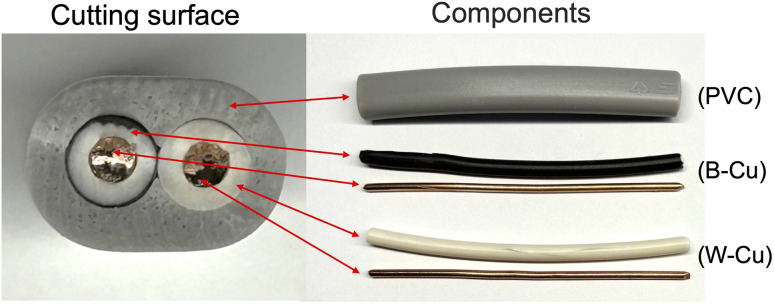
Photographs cutting surface of a two-core VVF cable and its components.Component: (PVC) polyvinyl chloride (PVC) covering, (B–Cu) black inner covering and copper wire, (W–Cu) white inner covering and copper wire.

We utilized a VVF power cable (VVF2X1.6; oval cutting surface = 6.0 mm × 8.7 mm) as a model for e-waste. This cable, obtained from Ohm Electric Inc., is a residential electric cable (see Fig. 1) with a two-core structure bundled together in a soft polyvinyl chloride covering (PVC; average degree of polymerization, 1300; see (PVC) in Fig. 1). The internal copper wire (diameter, 1.6 mm) is encased in a black (see B–Cu in Fig. 1) or white (see W–Cu in Fig. 1) covering with a diameter of approximately 3.2 mm.

**Fig. 2:**
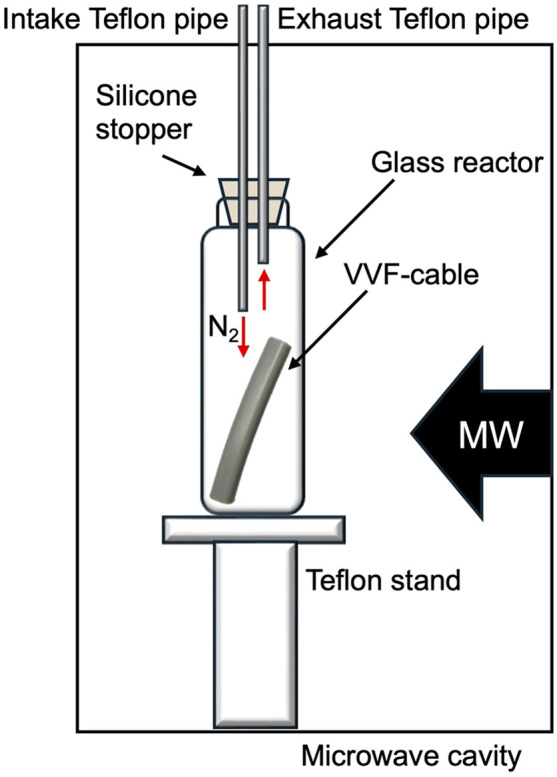
Illustrative image of a microwave cavity with a reactor containing a VVF cable.

The microwave irradiation equipment used was a Wave Magic MWO-1000s model, manufactured by Tokyo Rikakikai Co., Ltd. The image in Fig. 2 displays the microwave cavity and the setup of the glass reactor and VVF cable. The cut VVF cable was placed in a glass reactor (*φ*40 × 120 mm) and positioned in the microwave cavity. A silicone plug with two Teflon pipes (inner diameter 3 mm) was used to cover the top of the reactor, with the Teflon pipes extending from the top of the microwave cavity. Nitrogen gas was introduced into the reactor using a intake Teflon pipe in Fig. 2 and to prevent combustion from the pyrolysis process, and a exhaust pipe in Fig. 2 was used to allow gas to exhaust into the atmosphere. The microwave-assisted pyrolysis process was conducted at atmospheric pressure, and the position of the glass reactor was adjusted using a Teflon stand to align with the microwave irradiation port.

The pyrolysis behaviour of the VVF cable was studied by varying the microwave power and irradiation time. Samples of the VVF cable with lengths of approximately 1, 6, 9, 12, and 18 cm were used. Additionally, a 54 cm VVF cable was placed in a glass reactor (*φ*40 × 120 mm) and exposed to microwave irradiation under a nitrogen atmosphere.

In a separate experiment, pyrolysis of a VVF cable under reduced pressure was conducted using microwave irradiation. A pump was connected to the intake Teflon pipe in Fig. 2 while the exhaust Teflon pipe in Fig. 2 was sealed). The reduced pressure was set to about 100 Pa.

### 2.2 Simulation and analysis equipment

The electric field within the microwave cavity was analysed using a 3D FEM simulation software tool called Femtet by Murata Software Co. Ltd. The simulation involved studying changes in electric field strength using 2.45 GHz microwaves with pyrolysis occurring at an ambient temperature of 25 °C. The simulation space matched the dimensions (180(*W*) × 180(*D*) × 250(*H*) mm) of the actual microwave cavity used in the experiment. The material constants of the copper wire were as follows: (a) thermal conductivity: 402 W m^−1^ K^−1^, (b) electrical conductivity: 5.97 × 10^7^ S m^−1^, (c) relative magnetic permeability: 1.0, and (d) relative dielectric constant: 1.0. On the other hand, the material constants of the soft PVC material covering were: (i) thermal conductivity: 0.147 W m^−1^ K^−1^, (ii) electrical conductivity: 0, (iii) relative magnetic permeability: 1.0, (iv) relative dielectric constant: 3.3, and (v) tan *δ*: 0.12. These material properties were obtained from the data provided in the Femtet software, while the relative dielectric constant and tan *δ* of the soft PVC were measured using the actual sample employed in the experiment.

The substances resulting from the VVF cable pyrolysis were analysed using multiple methods. The gas emitted from the exhaust Teflon pipe in Fig. 2 was bubbled into 30 mL of hexane or methanol, and the products were analysed using a gas chromatograph/mass spectrometer (Shimadzu Corporation, GCMS-QP2010). The pyrolyzed PVC covering material was soaked in hexane or methanol for one day, after which the analysis was conducted using GC/MS. The GC/MS settings were as follows: Rxi-5Sil MS (30 m × 0.25 mm, 0.25 μm), helium as the carrier gas (2.21 mL min^−1^), inlet temperature at 250 °C, and the ion source temperature at 200 °C. The substances were identified using the GC/MS database provided by Shimadzu Co. Furthermore, the pyrolyzed PVC covering material was also examined using FT-IR (JASCO Co. FT/IR-6000) and diamond-based ATR (JASCO Co. ATR PRO ONE) techniques.

## 3 Results and discussion

### 3.1 Characteristics of the microwave-induced pyrolysis of VVF cables

The VVF cables were cut into different lengths and then exposed to microwave heating. The progress of the pyrolysis was assessed through visual inspection. The criteria for visual inspection involved observing the softening and carbonization of a portion of the VVF cable, as well as changes in shape or charring. It was important to note that mere temperature increase was not considered as indicative of pyrolysis. The summarized results can be found in Table 1.

**Table 1:** Pyrolysis of 1–18 cm lengths of samples of VVF cables subjected to 100, 200, and 300 W microwave irradiation^a^

Length/cm	MW power/W	Irradiation time/s			
		10	30	60	120
1	100	No	No	No	No
	200	No	No	No	No
	300	No	No	No	No
3	100	No	No	No	No
	200	No	Yes	Yes	Yes
	300	No	Yes	Yes	Yes
6	100	No	No	No	No
	200	No	No	No	No
	300	No	No	No	No
9	100	No	No	Yes	Yes
	200	No	Yes	Yes	Yes
	300	No	Yes	Yes	Yes
12	100	No	No	No	No
	200	No	No	No	No
	300	No	No	No	No
18	100	Yes	Yes	Yes	Yes
	200	Yes	Yes	Yes	Yes
	300	Yes	Yes	Yes	Yes

a Pyrolysis is herein defined as deformation due to softening of the VVF cable or carbonization due to scorching; a “Yes” indicates carbonization, while a “No” indicates no deformation had occurred.

**Fig. 3:**
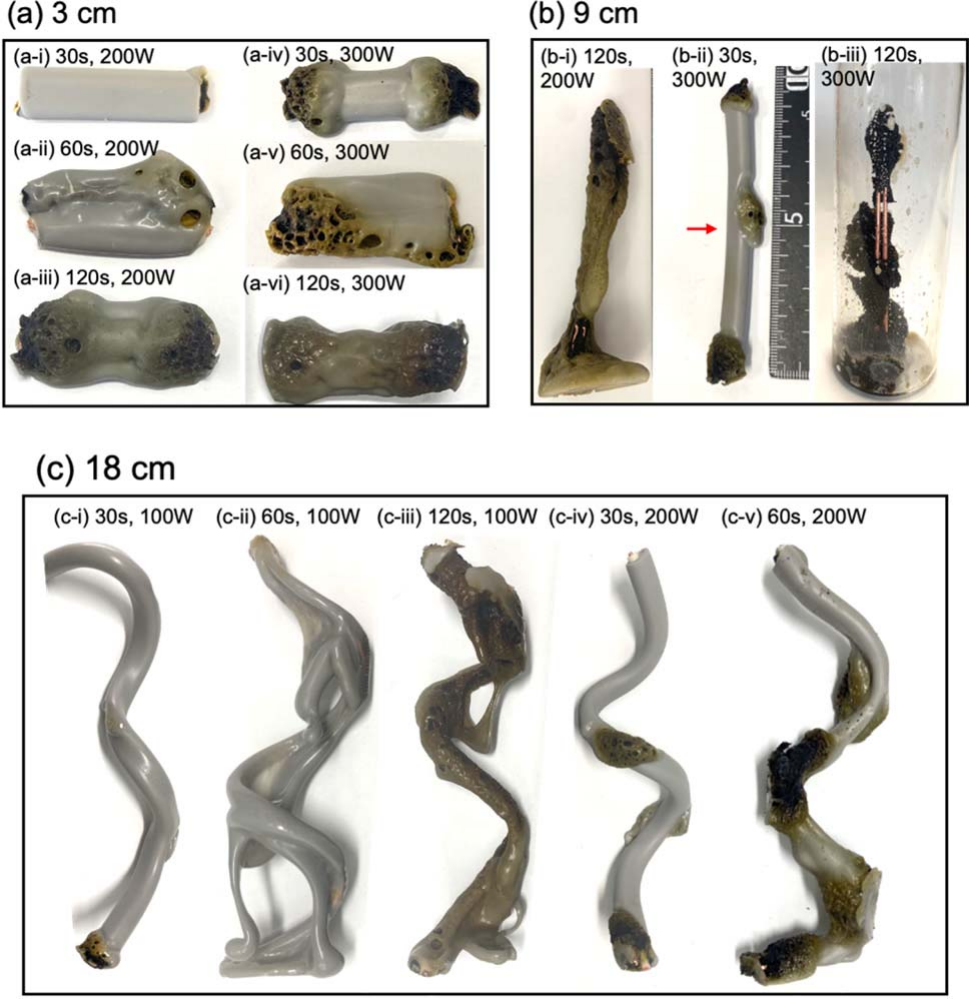
Photographs of VVF cables of various lengths pyrolyzed by microwave irradiation under different conditions: (a) 3 cm VVF cable length irradiated with 200 W or 300 W microwaves, (b) 9 cm VVF cable length irradiated with 200 W or 300 W microwaves, and (c) 18 cm VVF cable length irradiated with 100 W or 200 W microwaves.

Upon irradiating a 1 cm long VVF cable with 100 W microwaves, no changes were observed. Increasing the microwave output power to 200 W and 300 W also did not result in pyrolysis of the VVF cable. Subsequently, when the VVF cable sample was lengthened to 3 cm and irradiated with 100 W microwaves for up to 120 s, no visual changes were noted. However, raising the microwave output power to 200 W and irradiating for 30 s resulted in slight pyrolysis at the tip of the VVF cable, accompanied by the formation of white smoke (refer to Fig. 3a-i). Further irradiation for 60 s caused the entire VVF cable to soften, melt, and partly char and carbonize, generating a significant amount of white smoke (refer to Fig. 3a-ii). When the 3 cm sample was irradiated for 120 s, significant pyrolysis occurred from both ends of the VVF cable, with carbonization observed over a wide area (refer to Fig. 3a-iii). Additionally, using 300 W microwave irradiation of the VVF cable for 30 s led to notable pyrolysis, as evidenced by carbonization at both ends of the cable (refer to Fig. 3a-iv). Prolonged irradiation revealed pyrolysis progressing towards the center, accompanied by carbonization (refer to Fig. 3a-v and vi).

Based on the results, we hypothesized that microwaving VVF cables could lead to pyrolysis, but the extent of pyrolysis would vary depending on the length of the VVF cable. To test this hypothesis, we conducted a pyrolysis experiment with a 6 cm long VVF cable. Despite altering the microwave irradiation conditions, we observed no heat generation or pyrolysis as we did with the 1 cm cable. However, when a 9 cm VVF cable underwent pyrolysis *via* microwaves, pyrolysis occurred from both ends of the cable, similar to what we observed with the 3 cm cable. Furthermore, we observed pyrolysis with 60 s of irradiation using 100 W microwaves. Additionally, upon irradiation for 120 s with 200 W microwaves, vigorous thermal pyrolysis occurred in the glass container, causing drippings of the PVC covering at the bottom (Fig. 3b-i). With 300 W microwaves and 30 s of irradiation, pyrolysis occurred near the center (4.5 cm) in addition to both ends (Fig. 3b-ii). Moreover, after 120 s of microwave irradiation, the PVC covering decomposed aggressively, exposing a naked copper wire (Fig. 3b-iii). Interestingly, the 9 cm cable underwent more rapid pyrolysis compared to the 3 cm VVF cable under the same microwave output power and irradiation times (compare, for example, Fig. 3a-vi*versus*Fig. 3b-iii and Fig. 3a-iii with Fig. 3b-i).

**Fig. 4:**
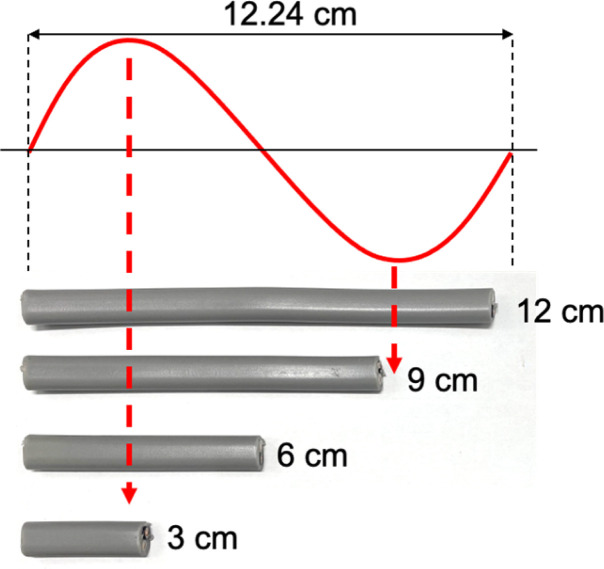
Illustrative connections of the VVF cable lengths and the microwave wavelength.

The previous observations suggest a correlation between the lengths of VVF cables and the wavelength of the microwaves, specifically those that are multiples of 3 cm. For instance, the wavelength of the 2.45 GHz microwaves used in this study was approximately 12.24 cm, and the VVF cable lengths at which pyrolysis occurred most vigorously were 3 cm and 9 cm. These lengths correspond to 1/4 of the wavelength (3.06 cm) and 3/4 of the wavelength (9.18 cm) respectively. It appears that the copper wires in the VVF cables at these specific lengths acted as receiving antennas for the microwaves, resulting in discharge and pyrolysis as illustrated in Fig. 4.

Based on this assumption, we would not anticipate pyrolysis to take place in a 12 cm VVF cable subjected to these microwave conditions, corresponding to the wavelength. As anticipated, there were no visual signs of pyrolysis in the 12 cm VVF cable, even when exposed to 300 W microwaves for 120 s the highest surface temperature recorded was only 52 °C. Therefore, the length of the VVF cable is a crucial factor in microwave-induced pyrolysis.

The following questions now arise: will pyrolysis of a VVF cable longer than the wavelength occur, and will the pyrolysis proceed even if the cable were bent? To address these two queries, an 18 cm VVF cable was spirally rolled to a height of 14 cm and exposed it to microwaves. Pyrolysis was observed at the end and in the middle of the spiral sample after irradiation with 100 W microwaves for 30 s (Fig. 3c-i). Subsequently, after 60 s of microwave exposure, the PVC covering was heated and softened overall (Fig. 3c-ii); after 120 s, pyrolysis and carbonization progressed further (Fig. 3c-iii). Similarly, when irradiated with 200 W microwaves, pyrolysis occurred at the end and at the center (9 cm) of the VVF cable after 30 s of exposure, with carbonization also observed at those positions (Fig. 3c-iv). Partial dissolution and carbonization due to pyrolysis were noted after 60 s of microwave irradiation (Fig. 3c-v). These observations indicate that at low microwave power outputs, the entire PVC covering of the VVF cable softened overall, accompanied by overall carbonization. Conversely, an increase in microwave power caused partial heating of the VVF cable, as well as partial dissolution and carbonization of the PVC covering.

**Fig. 5:**
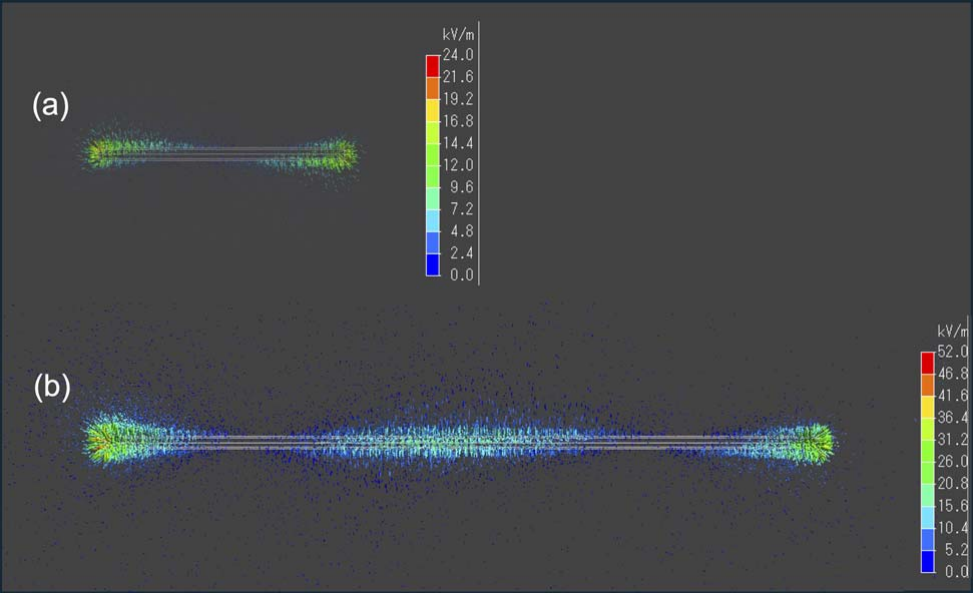
Images of the electric field intensity (kV m^−1^) simulation for (a) 3 cm and (b) 9 cm VVF cable lengths.

The findings depicted in Fig. 3 prompted us to explore another aspect. When analyzing the 9 cm copper wire, we observed pyrolysis occurring not only at both ends but also near the center. Conversely, with the 3 cm wire, no pyrolysis was visually observed at the center. This suggests that the microwave electric field of the 9 cm copper wire was concentrated at both ends and near the center, leading to pyrolysis in these areas. Consequently, we conducted an electromagnetic field analysis simulation to calculate the electric field strength in a VVF cable a two-core copper wire used in a microwave cavity of the same size as the experiment. For the 3 cm copper wire, the electric field was concentrated only at both ends (Fig. 5a), while for the 9 cm wire, it was concentrated at both ends and at the center (Fig. 5b). This indicates that these electric field concentrations caused localized pyrolysis, especially when the microwave power was increased to 300 W. Additionally, the electric field strength at 9 cm was, on average, twice as high as at 3 cm. For instance, a comparison between Fig. 3a-vi and b-iii suggests that the reason for the more rapid pyrolysis at 9 cm than at 3 cm, even under identical microwave power, is likely due to the concentration of electric field strength.

### 3.2 Mechanistic implications in microwave-induced pyrolysis

Based on our thorough observations during the experiment and the resulting data, we hypothesized that the pyrolysis of VVF cable when exposed to microwaves would occur either simultaneously or independently due to (1) heat from discharge and (2) heat from microwave dielectric and induction heating. To test these hypotheses, we carried out individual experiments.

**Fig. 6:**
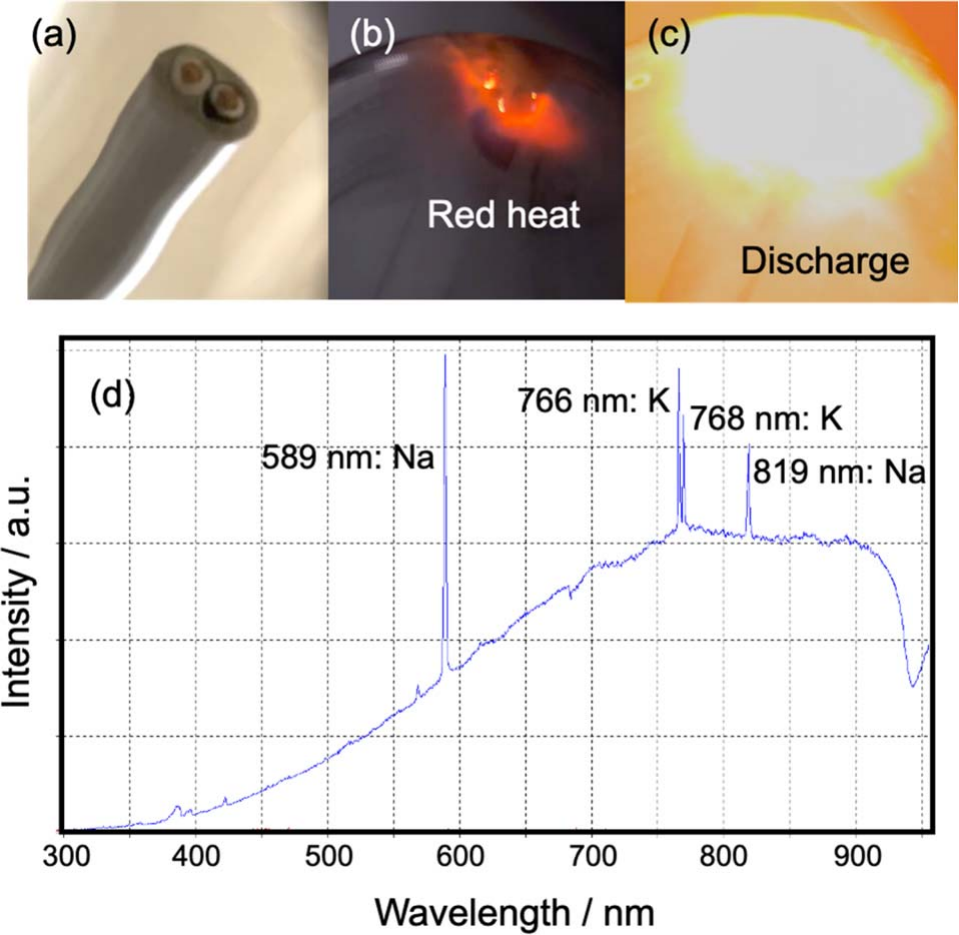
Photographs and spectral analysis of plasma generation at the tip of a VVF cable under microwave irradiation. (a) Initial VVF cable tip, (b) VVF cable tip glowing red, (c) VVF cable tip discharging, (d) UV-Vis spectral features of the emitted radiation generated from the white-orange light emitted from the end of the VVF-cable under microwave irradiation.

(1) Heat originating from a discharge: throughout the experiment, we frequently observed red and white-orange light emissions in the copper wires of the 3, 9, and 18 cm VVF cables. This led us to conclude that pyrolysis was taking place in the VVF cables due to microwave irradiation causing a discharge and plasma generation. To further analyze the discharge observed under 300 W microwave irradiation, we placed the 6 and 12 cm VVF cables in a quartz reactor and conducted emission spectroscopy. Interestingly, no light was emitted when the 6 cm VVF cable was irradiated with microwaves for 120 s. However, the 9 cm VVF cable (Fig. 6a) exhibited red light emission near the copper wire immediately after microwave irradiation (Fig. 6b), followed by a white-orange light emission, which we attributed to an arc discharge (Fig. 6c).

The UV-visible emission of the white-orange glow coming from the VFF cable end was observed in the 300–950 nm wavelength range using a fiber optic cable (positioned at a fixed distance of 10.0 cm from the lamp) connected to a high-sensitivity UV-Vis Spectrophotometer (Ocean Optics Inc., Maya2000Pro). The emission spectrum exhibited 589 nm and 819 nm spectral lines associated with sodium (Na), as well as 766 nm and 768 nm attributable to potassium (K).^15^ Typically, white light corresponds to a temperature greater than approximately 1400 °C, while the temperature of the orange light is around 930 °C.^16^ Thus, we hypothesize that the copper wire in the VVF cable reached such temperatures when exposed to the electric field of microwaves. According to Yu *et al.*,^17^ the dehydrochlorination of PVC primarily occurs at low temperatures of 250–320 °C from the PVC insulation used in power cables. Temperatures significantly above 350 °C lead to the breakdown of carbon chains into smaller molecules, ultimately resulting in carbonization, as the threshold temperature for PVC decomposition is much lower than that of other plastics. Note that no melting was observed in the copper wire exposed to high temperatures, as the melting point of copper is 1085 °C,^18^ and even if a discharge were to occur, it would likely be in a temperature range below that.

The study also investigated the occurrence of microwave discharge in the 6 and 12 cm cables. It is a well-established fact that generating a microwave spark discharge at low power levels requires reducing the pressure inside the glass container in accordance with Paschen's law.^19^ Accordingly, a pump was attached to the intake Teflon pipe in Fig. 2 (while the exhaust Teflon pipe was sealed) to lower the pressure to 100 Pa, followed by exposing the samples to microwave powers of 100, 200, and 300 W. To provide a point of comparison, an experiment was also conducted on a 3 cm long VVF cable under reduced pressure of 100 Pa. The findings are summarized in Table 2.

**Table 2:** Presence or absence of microwave pyrolysis in VVF cables cut to lengths of 3 to 12 cm under reduced pressure of 100 Pa. Presence or absence of microwave pyrolysis in only the PVC covering of VVF cables under atmospheric pressure^a^

Length/cm	MW power/W	Irradiation time/s			
		10	30	60	120
3 (decompression: 100 Pa)	100	No	No	Yes	Yes
	200	No	Yes	Yes	Yes
	300	No	Yes	Yes	Yes
6 (decompression: 100 Pa)	100	No	No	No	Yes
	200	No	Yes	Yes	Yes
	300	No	Yes	Yes	Yes
12 (decompression: 100 Pa)	100	No	No	No	Yes
	200	No	No	Yes	Yes
	300	No	No	Yes	Yes
3 (PVC covering alone)	100	No	No	No	No
	200	No	No	No	No
	300	No	No	No	No
6 (PVC covering alone)	100	No	No	No	No
	200	No	No	No	No
	300	No	No	Yes	Yes
9 (PVC covering alone)	100	No	No	No	No
	200	No	No	Yes	Yes
	300	No	No	Yes	Yes
12 (PVC covering alone)	100	No	No	No	No
	200	No	No	Yes	Yes
	300	No	No	Yes	Yes

a Pyrolysis is defined as VVF cable deformation due to softening or carbonization due to scorching; a “Yes” indicates deformation while a “No” indicates no deformation.

**Fig. 7:**
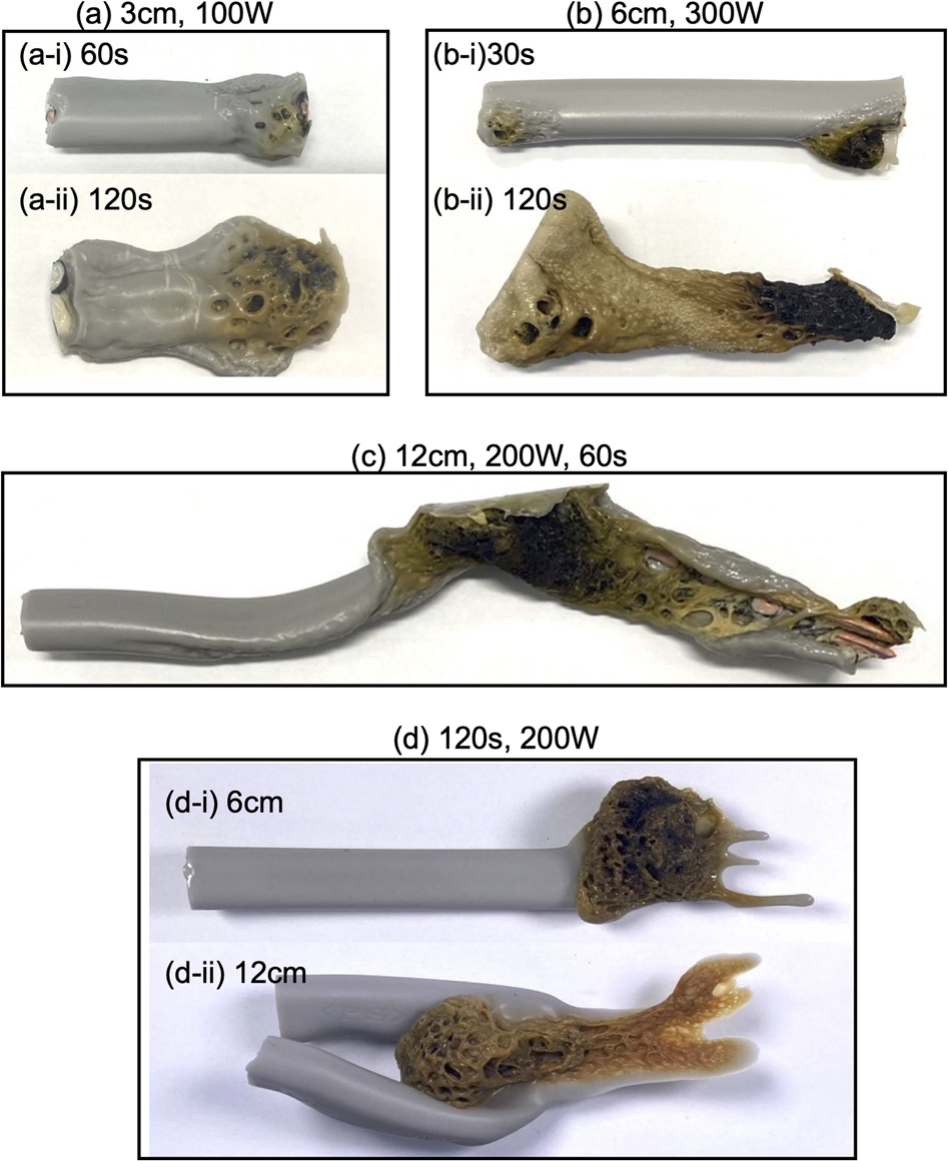
Photographs of the pyrolysis of VVF cable by microwave irradiation under various conditions. (a) 3 cm VVF cable length irradiated with 200 and 300 W microwaves, (b) 9 cm VVF cable length irradiated with 200 and 300 W microwaves, (c and d) 18 cm VVF cable length irradiated with 100 and 200 W microwaves.

A discharge and pyrolysis were observed coming from the tip of the VVF cable when a 3 cm long VVF cable was exposed to 100 W microwaves for 60 s under reduced pressure (approximately 100 Pa) (see Fig. 7a-i). The experiment confirmed that reducing the pressure enhanced the microwave discharge generation efficiency, as pyrolysis did not occur under atmospheric pressure (refer to Table 1). Additionally, when the VVF cable was irradiated with microwaves for 120 s, the PVC covering softened and underwent pyrolysis (see Fig. 7a-ii).

A VVF cable measuring 6 cm in length (see Table 1) did not undergo microwave-induced pyrolysis under ambient pressure conditions. However, with microwave irradiation under reduced pressure, red heat and a discharge were observed from both ends of the cable after a 30 second irradiation period (refer to Fig. 7b-i). After 120 s, the entire PVC covering had completely softened and melted off (see Fig. 7b-ii). In the case of a 12 cm long VVF cable, pyrolysis progressed after a 60 s exposure to 200 W microwaves under reduced pressure conditions, resulting in significant softening and carbonization of the PVC covering material (see Fig. 7c). These findings strongly indicate that the pyrolysis of the VVF cable is influenced by its length and the microwave wavelength. This dependence suggests the ease with which pyrolysis due to discharge happens, even if the cable is not of the specific length, under reduced pressure.

The work function of copper ranges from 4.53 to 5.10 eV,^20^ which means that electrons are not released without a significant amount of energy. Consequently, VVF cables are less likely to cause microwave discharges. However, reducing the pressure can easily lead to discharges. In any case, increasing microwave power to 600 W under atmospheric pressure resulted in discharges from the ends of 6 cm and 12 cm VVF cables. Therefore, the length of the VFF cable is a significant factor in inducing microwave discharges.

Research on pyrolysis is commonly carried out under an inert nitrogen atmosphere. However, the costs associated with using this gas can hinder the economic feasibility and scalability of the process.^21^ Alternatively, conducting pyrolysis under reduced pressures could lead to a more cost-effective process. At lower pressures, pyrolysis has shorter residence times, yields greater liquid product, and is more energy-efficient due to lower decomposition temperatures.^22^ Therefore, the use of the microwave method to extract valuable quantities of copper from power VVF cables through pyrolysis under reduced pressures makes it a more feasible option for potential scale-up.

(2) Microwave dielectric and induction heating: here, we present an additional, potentially alternative explanation for heat generation in the absence of discharges.

(a) Pyrolysis of PVC coverings by microwave heating: the PVC covering shown in Fig. 1, from which the two inner copper wires with black and white coverings (B–Cu and W–Cu in Fig. 1) were removed, was subjected to microwave pyrolysis. Interestingly, no pyrolysis was observed for the 3 cm PVC covering even under 300 W microwave irradiation (refer to Table 2). However, pyrolysis did occur for the 6 cm VVF cable when exposed to 300 W microwaves for 60 s (Fig. 7d-i). Under the conditions specified in Table 1, no pyrolysis was observed for the 6 cm VVF cable containing the copper wires, although the absence of the copper wire (solely for the PVC covering) did result in the generation of white smoke, indicating the occurrence of pyrolysis. Moreover, pyrolysis also occurred for the 9 cm cable when exposed to 200 W microwaves for more than 60 s. This trend was similarly observed for the 12 cm cable (Fig. 7d-ii). Thus, microwaves can directly induce pyrolysis in the PVC covering of the VVF cable.

The study also included the measurement of the dielectric factor of PVC covering material to examine its heating efficiency when exposed to microwaves. The dielectric constant of the VVF cable containing copper wires was found to be *ε*′ = 3.24, with a dielectric loss of *ε*′′ = 0.38. In contrast, the dielectric constant of the PVC covering material alone was also 3.24, but with a lower dielectric loss of 0.34, indicating a decrease when the copper wires were removed. The dielectric factor of ion-exchanged water was significantly higher, with a dielectric constant of 77.4 and a dielectric loss of 9.35, suggesting a lower heating efficiency compared to water. Moreover, when the PVC covering material was heated to 50 °C and 70 °C in an electric furnace, its dielectric constant and dielectric loss were measured. At 50 °C, the dielectric constant was 3.34 and the dielectric loss was 0.43, while at 70 °C, the dielectric constant was 3.31 and the dielectric loss was 0.49. This indicates that microwave heating of PVC covering material is not efficient at room temperature but becomes easier at higher temperatures.

**Fig. 8:**
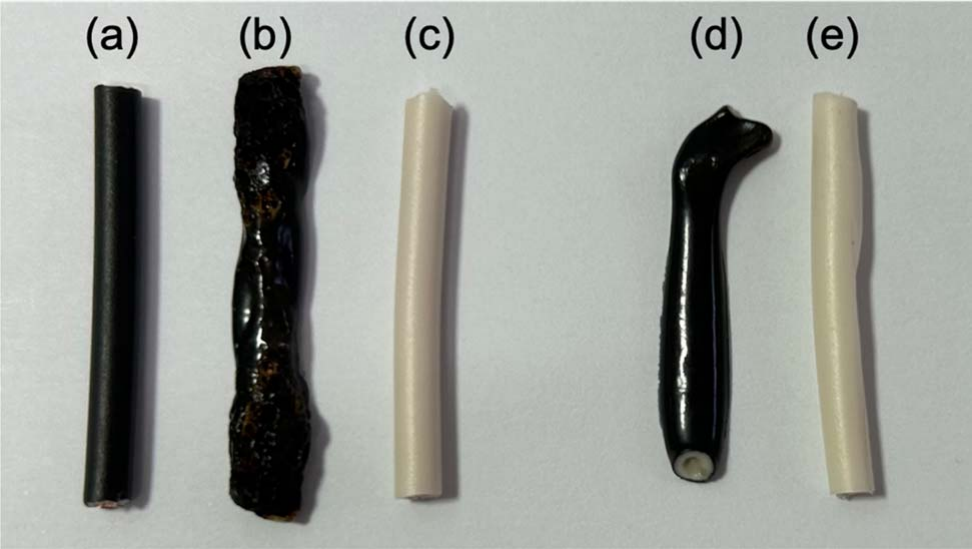
Photographs of the pyrolysis of the covering material with copper wires (3 cm) and without copper wires (3 cm) exposed to 300 W microwave irradiation. (a) Black-coated copper wire before microwave irradiation, (b) black-coated copper wire after 30 s of microwave irradiation, (c) white-coated copper wire after 300 s of microwave irradiation, (d) black covering without copper wire after 180 s of microwave irradiation, (e) white covering without copper wire after 300 s of microwave irradiation.

(b) Pyrolysis of copper wire covering by microwave heating: in the next experiment, we examined the effectiveness of using microwave heating to conduct the pyrolysis of the copper wire covering within the PVC. The heating trials were conducted using 300 W microwaves on both black and white cables containing copper wires, as well as on the covering alone, as shown in B–Cu and W–Cu in Fig. 1. The microwave irradiation process involved placing a 9 cm diameter glass Petri dish on a Teflon stand inside the microwave cavity (refer to Fig. 2). Subsequently, a black and a white cable were positioned in parallel within the dish, and microwave radiation was activated. Photographs capturing the morphological changes of the samples after irradiation can be seen in Fig. 8.

The black cable's surface with the copper wire (Fig. 8a) melted immediately upon exposure to microwave radiation, resulting in some carbonized areas due to pyrolysis after 30 s (Fig. 8b). In contrast, no change in shape or color was observed for the white cable, despite also containing 3 cm of copper wire. The white cable showed no signs of pyrolysis even after 300 s of microwave irradiation (Fig. 8c). To investigate why only the black cable produced heat, we microwaved the cable without the copper wire. After 180 s of exposure to 300 W microwaves, the surface of the cable melted and exhibited signs of pyrolysis (Fig. 8d). In contrast, the white cable showed no changes even after 180 s of microwave irradiation, and no changes were observed after an additional 300 s (Fig. 8e). These findings suggest that the black cable's insulation of the copper wire likely contains a chemical component that induces microwave heating, possibly related to the presence of carbon black for coloring. Previous reports have indicated that carbon black in rubber can generate an ultra-high temperature field due to interfacial polarization.^23^ However, although the heat generation rate of the black covering material may not be fast, it is not the rate-limiting factor for the pyrolysis of the VVF cable.

### 3.3 Analysis of microwave pyrolysis products

The chemical products resulting from the microwave pyrolysis of VVF cable were examined using GC/MS techniques. In this study, a 9-cm VVF cable was exposed to 300 W microwaves. The gas emitted from the exhaust Teflon pipe in Fig. 1 was passed through a hexane or methanol solvent for dissolution and subsequent analysis. The small amounts of 2-hexenal (C_6_H_10_O), 3-methyl-1-hexanol (C_7_H_16_O), 6-methylheptanol (C_8_H_18_O), 1-nonanol (C_9_H_20_O), phthalic anhydride (C_8_H_4_O_3_) and octamethylcyclotetrasiloxane (C_8_H_24_O_4_Si_4_) were producted. The production of HCl was observed during the initial stage of pyrolysis under microwave irradiation, leading to the rapid dechlorination of the PVC covering material. Subsequent stages involved the breakdown of polymer chains, resulting in the noted hydrocarbon compounds. Phthalic anhydride serves as a chemical intermediate in the manufacture of plasticizers from polyvinyl chloride (PVC). In the meantime, octamethylcyclotetrasiloxane serves as an impurity in silicone resin that is incorporated into the covering material for its heat-resistant and electrical insulation properties; its high volatility suggests that it is likely gasified during pyrolysis.

**Fig. 9:**
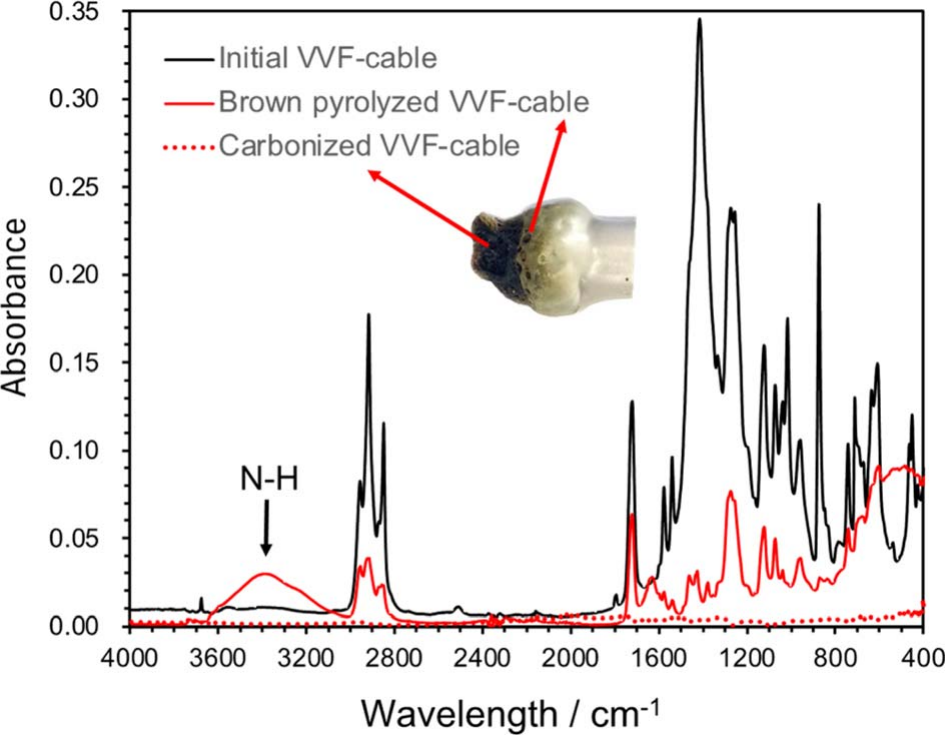
ATR spectrum of PVC covering material during pyrolysis of VVF cable (initial PVC covering material, brown discolored area or carbonized area due to microwave pyrolysis).

Microwaves pyrolysis process might occur in short time during which any intermediate formed might be carbonized promptly, thereby suppressing the formation of tar-like substances, polycyclic aromatic compounds, or dioxins as none were detected subsequent to the dechlorination. Nevertheless, the discolored sections of the VVF cable were treated with hexane or methanol to extract the components for analysis using GC/MS methods. The analysis revealed the presence of additional substances produced during the pyrolysis process, namely: 6-methylheptanol (C_8_H_18_O), 1-nonanol (C_9_H_20_O), 7-methyl-1-undecene (C_12_H_24_), and 1-chloroundecane (C_11_H_23_Cl). Note that these substrates were absent in the original VVF cable. Next, ATR analysis using FT-IR was conducted on the browned and carbonized parts of the PVC coating of the VFF cable after pyrolysis (Fig. 9). The ATR analysis of the browned PVC coating revealed a reduction in the peaks associated with PVC and additives, such as plasticizers. Conversely, a new broad peak appeared around 3600–3200 cm^−1^, which is believed to correspond to N–H stretching, likely formed due to microwave irradiation in a nitrogen atmosphere. In contrast, no significant peaks were observed in the carbonized sample, suggesting that its main component is carbon.

### 3.4 Carbonization of VVF cable by microwave-induced pyrolysis and recovery of copper

**Fig. 10:**
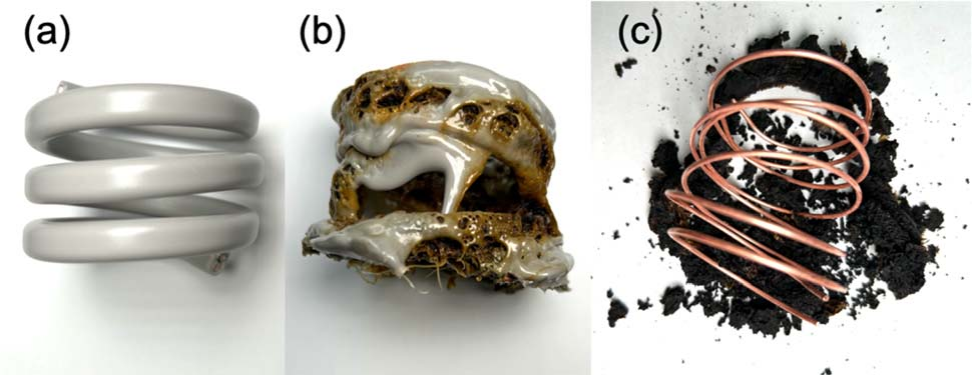
Photographs of carbonization of PVC covering and recycling of copper wire during the pyrolysis of a 54 cm spring-shaped VVF cable. (a) Initial spring-shaped VVF cable, (b) after irradiation with 300 W microwaves for 3 min, and (c) irradiation with 300 W microwaves for a 12 min period.

We further conducted a study to determine if microwave radiation could carbonize the insulation of the VVF cable and enable the complete removal of the copper wires through carbonization, making it easier to recover the copper metal. We utilized a 54 cm VVF cable (Fig. 10a) wound into a spring shape (3.5 turns, outer diameter 5.5 cm, inner diameter 4.7 cm, height 4.5 cm), which was then placed in a glass reactor (*φ*120 × 160 mm) and exposed to 300 W microwaves under nitrogen at ambient pressure. We monitored dynamic changes in pyrolysis time during the process.

The microwave irradiation initiated the production of white smoke while causing the 54 cm cable to undergo pyrolysis. The covering material began melting off the coil after the microwave irradiation began. Within 4 min, most of the PVC covering material had melted, revealing carbonization inside the cable (Fig. 10b). This suggests that the area near the copper wire was heated by the microwaves, leading to heating progression within the VVF cable. After 12 min of microwave irradiation, the carbonized covering material peeled off from the copper wiring (Fig. 10c). Although some carbonized material remained around the copper wiring, it fell off completely when removed from the reactor, leaving behind a clean copper wire. No significant visible discharge occurred during the 12 min microwave irradiation, and there were no observable discharges during the pyrolysis. The copper wire was continually exposed throughout the process. No signs of melting or damage were observed in any part of the copper wire, a welcomed outcome for promoting a recycling process.

The carbonized samples were subsequently subjected to elemental analysis using energy dispersive X-ray fluorescence spectroscopy (ED-XRF; Shimadzu Co., EDX-8100). Apart from carbon, the primary element detected was Ca, which is an additive found in the PVC covering material. This additive was also present in the original PVC covering material. As well, a small amount of Cl was detected, indicating the presence of some non-dechlorinated chloride component. Additionally, elements such as Al, Na, K, Si, S, Cu, Zn, Fe, Cr, and Sr were also identified and whose presence was less than 1%, which accorded with the findings from a spectroscopic analysis of a pristine VVF cable.

## 4 Concluding remarks

The pyrolysis of VVF cables induced by microwaves studied in detail. The initial phase involves heating due to a discharge near the copper wire. Interestingly, when the length of a copper wire is 1/2 or 1/1 of the microwave wavelength, no discharge occurs under weak microwave irradiation conditions, unless the microwave power is increased or the pressure is reduced. Moreover, VVF cables longer than a wavelength do not have discharge limitations, making uncut VVF cables more suitable for microwave pyrolysis than cut ones, which offers significant advantages for recycling. When the covering material is carbonized by the discharge, it becomes a microwave absorber.

Apart from electrical discharge, the covering material was progressed by the microwave heating directly. This direct application of microwaves boosts the efficiency of microwave heating as temperatures rise, leading to increased dielectric loss. For even greater efficiency, the pyrolysis process can be further improved by initially pre-heating the cable with a conventional heater.

In a study on the microwave pyrolysis of PVC, researchers found that the addition of a microwave absorbent, such as activated carbon or ferrite, was necessary to effectively remove hydrogen chloride from PVC, even when using a 500 W commercial microwave oven.^24^ This led to the suggestion that e-wastes containing metals is better suited for microwave pyrolysis than separated e-wastes, such as separated insulation and copper wire. Since pyrolysis occurred rapidly, only small amounts of decomposition products were produced as the covering materials were quickly carbonized.

Furthermore, the process yields some scale-up results: discharge serves as a critical factor in microwave pyrolysis. However, it's worth noting that microwave discharge can also potentially lead to fire under certain circumstances.^25^ To address this issue, the use of inert gas or reduced pressure has proven effective. Nonetheless, it's important to consider that reduced pressure can facilitate the generation of electrical discharges, thereby offering a cost-effective solution for the overall process.

## Data availability

All data used in this paper are unpublished and all original. Raw data were generated at corresponding author (S. H.).

## Author contributions

S. H. designed and guided this project. N. H. and S. H. conducted the experiments. S. H. wrote the first draft, which was subsequently re-examined following various discussions regarding the data's interpretation by N. S. and S. H.

## Conflicts of interest

There are no conflicts of interest to declare.

## References

[cit1] See: https://www.globalewaste.org

[cit2] Cole C., Gnanapragasam A., Cooper T., Singh J. (2019). Assessing barriers to reuse of electrical and electronic equipment, a U.K. perspective. Resour. Conserv. Recycl..

[cit3] Toral-López V., González C., Romero F. J., Castillo E., Parrilla L., García A., Rodriguez N., Rivadeneyra A., Moralesa D. P. (2018). Reconfigurable electronics: Addressing the uncontrolled increase of waste electrical and electronic equipment. Resour. Conserv. Recycl..

[cit4] See: https://www.ewastemonitor.info/the-global-e-waste-monitor-2024/

[cit5] Rene E. R., Sethurajan M., Kumar Ponnusamy V., Kumar G., Bao Dung T. N., Brindhadevi K., Pugazhendhi A. (2021). Electronic waste generation, recycling and resource recovery: technological perspectives and trends. J. Hazard. Mater..

[cit6] Joshi S., Sharma M., Das R. P., Muduli K., Raut R., Narkhede B. E., Shee H., Misra A. (2022). Assessing effectiveness of humanitarian activities against COVID-19 disruption: the role of blockchain-enabled digital humanitarian network (BT-DHN). Sustainability.

[cit7] Ikhlayel M. (2017). Environmental impacts and benefits of state-of-the-art technologies for E-waste management. Waste Manage..

[cit8] Sharma M., Luthra S., Joshi S., Kumar A. (2022). Developing a framework for enhancing survivability of sustainable supply chains during and post-COVID-19 pandemic. Int. J. Logist. Res. Appl..

[cit9] Wędrychowicz M., Kurowiak J., Skrzekut T., Noga P. (2023). Recycling of electrical cables – current challenges and future prospects. Mater.

[cit10] Sasaki S. (2000). Latest trends in PVC recycling technology. Plastics.

[cit11] Murata K., Aiba K., Ooya S., Tominaga Y., Matsumoto T., Mizuno K., Motomiya H. (2002). Development of insulated wire and cable using recycled PVC. Furukawa Rev..

[cit12] Enomoto H., Hatakeyama A., Kato Y. (1995). Dechlorination treatment of polyvinyl chloride. J. Jpn. Soc. Waste Manage. Experts.

[cit13] Andersson M., Knutson Wedel M., Forsgren C., Christéen J. (2012). Microwave assisted pyrolysis of residual fractions of waste electrical and electronics equipment. Miner. Eng..

[cit14] Risco A., Sucunza D., González-Egido S. (2021). Chemical recovery of waste electrical and electronic equipment by microwave-assisted pyrolysis: a review. J. Anal. Appl. Pyrolysis.

[cit15] MIT Wavelength Tables, Wavelength by Elements, ed. F.M. Phelps III, MIT Press, Boston, MA, 2nd edn, 1982, ISBN 9780262160872

[cit16] See: *e.g.*: https://www.en.wikipedia.org/wiki/Thermal_radiation

[cit17] Yu J., Ma C., Qiao Y., Qiao Y., Yao H. (2016). Thermal degradation of PVC: a review. Waste Manage..

[cit18] See *e.g.*, https://en.wikipedia.org/wiki/Copper

[cit19] Paschen F. (1889). Ueber die zum Funkenübergang in Luft, Wasserstoff und Kohlensäure bei verschiedenen Drucken erforderliche Potentialdifferenz. Ann. Phys..

[cit20] See: https://en.wikipedia.org/wiki/Work_function

[cit21] Zhu J., Chen X., Yao Z., Yin Y., Lin K., Liu H., Huang J., Ruan J., Qiu R. (2019). Directional concentration of bromine from nonmetallic particles of crushed waste printed circuit boards by vacuum-gasification-condensation. J. Cleaner Prod.c.

[cit22] Wan Mahari W. A., Chong C. T., Lam W. H., Anuar T. N. S. T., Ma N. L., Ibrahim M. D., Lam S. S. (2018). Microwave co-pyrolysis of waste polyolefins and waste cooking oil: influence of N_2_ atmosphere *versus* vacuum environment. Energy Convers. Manage..

[cit23] Okumura K., Saeki A., Hojo M., Takizawa T., Horikoshi S. (2022). Elucidation of the principle of microwave rubber vulcanization based on dielectric parameters and vulcanization of tire rubber by variable frequency microwave. J. Appl. Polym. Sci..

[cit24] Moriwaki S., Machida M., Tatsumoto H., Otsube Y., Aikawa M., Ogura T. (2006). Dehydrochlorination of poly(vinyl chloride) by microwave irradiation. Appl. Therm. Eng..

[cit25] Horikoshi S., Arai Y., Ahmad I., DeCamillis C., hicks K., Schauer B., Serpone N. (2020). Application of Variable Frequency Microwaves in Microwave-Assisted Chemistry: Relevance and Suppression of Arc Discharges on Conductive Catalysts. Catalysts.

